# Time-Normalization Approach for fNIRS Data During Tasks with High Variability in Duration

**DOI:** 10.3390/s25061768

**Published:** 2025-03-12

**Authors:** Anna Falivene, Charlotte Johnson, Katrijn Klingels, Pieter Meyns, Evi Verbecque, Ann Hallemans, Emilia Biffi, Caterina Piazza, Alessandro Crippa

**Affiliations:** 1 Scientific Institute IRCCS E. Medea, 23842 Bosisio Parini, Italy; emilia.biffi@lanostrafamiglia.it (E.B.); caterina.piazza@lanostrafamiglia.it (C.P.); alessandro.crippa@lanostrafamiglia.it (A.C.); 2 Research Group MOVANT, Department of Rehabilitation Sciences and Physiotherapy (REVAKI), University of Antwerp, 2610 Wilrijk, Belgium; charlotte.johnson@uantwerpen.be (C.J.); ann.hallemans@uantwerpen.be (A.H.); 3 Research Centre (REVAL), Faculty of Rehabilitation Sciences and Physiotherapy, Hasselt University, 3590 Diepenbeek, Belgium; katrijn.klingels@uhasselt.be (K.K.); pieter.meyns@uhasselt.be (P.M.); evi.verbecque@uhasselt.be (E.V.)

**Keywords:** data time-normalization, functional near-infrared spectroscopy, spline interpolation, self-paced tasks, MATLAB

## Abstract

Functional near-infrared spectroscopy (fNIRS) is particularly suitable for measuring brain activity during motor tasks, due to its portability and good motion tolerance. In such cases, the trials’ duration may vary depending on the experimental conditions or the participant’s response, therefore a comparison of hemodynamic responses across repetitions cannot be properly performed. In this work, we present a MATLAB (R2023a) function (*TaskNorm.m*) developed for time-normalizing fNIRS data recorded during trials with different durations. It is based on a spline interpolation method that rescales the time -axis to the percentage of the trial with a fixed number of samples. This allows us to successively average across repetitions to obtain the mean hemodynamic responses and complete the standard data processing. The algorithm was tested on eight subjects (four with developmental coordination disorder, age: 9.78 ± 0.30 and four typically developing children, age: 9.02 ± 0.30) performing three different tasks. The results show that the *TaskNorm* function works as expected, allowing both a comparison and averaging of the data across multiple repetitions. The performance of the function is independent of the task or the pre-processing pipeline applied. The proposed function is publicly available and importable into the HomER3 package (v1.72.0), representing a further step in the ongoing standardization process of fNIRS data analysis.

## 1. Introduction

In the last decades, the use of functional near-infrared spectroscopy (fNIRS) has seen an important increase as a tool for investigating functional brain activity with a wide range of applications in the field of neuroscience [1,2]. Functional NIRS is an optical neuroimaging technique that monitors hemodynamic changes within the brain through optical sensors placed on the surface of the scalp. In particular, light at different wavelengths (typically between 650 and 850 nm) is emitted by sources onto the surface of the head. Modifications in the optical absorption are then recorded by detectors to measure changes in blood oxygenation in terms of cortical oxyhemoglobin (ΔHbO_2_), deoxyhemoglobin (ΔHbR), and total hemoglobin (ΔHbT) concentrations. During evoked activity in the cortex of the brain, the increase in the blood flow in the active region alters the concentrations of the oxygenated and deoxygenated hemoglobin in the brain, resulting in changes in the absorption of light detected by the optodes [3]. Therefore, an increase in HbO_2_ and HbT and a corresponding decrease in HbR are expected in the activated areas [4,5].

Thus, fNIRS allows us to estimate brain activity indirectly based on hemodynamic changes in the brain, providing a valid alternative to functional magnetic resonance imaging (fMRI), which is considered the gold standard methodology for the assessment of cortical and subcortical activity [6]. Nonetheless, fMRI suffers from some important limitations related to its costs, sensitivity to movement artefacts, and the restricted range of motion in the scanner that affects both the tolerability of the technique and the types of tasks that can be performed [7].

Although each available neuroimaging technique (e.g., fMRI, electroencephalography-EEG, magnetoencephalography, and positron emission tomography) has advantages and disadvantages, when compared to others, fNIRS is low-cost, non-invasive, and portable, with a relatively good spatial/temporal resolution, and a good tolerance to motion [8]. Additionally, fNIRS is particularly suitable to monitor brain activity in clinical and pediatric populations, which traditionally experience more difficulties in undergoing fMRI [9]. Consequently, fNIRS has been classically exploited to assess task-related cortical activation during both cognitive (e.g., picture identification [10]; emotion recognition [11,12]; and memory tasks [13]), and motor activities [1] (e.g., walking [14,15]; balance control tasks [16,17] involving walking over obstacles [18,19], and stepping tasks [20]), as well as activities involving dual tasks (typically walking with a concomitant cognitive and/or motor task [21,22]).

Experimental fNIRS protocols commonly include a baseline period and tasks with fixed durations, but in some cases, the trials’ duration may vary depending either on the experimental conditions or the participant’s response. This circumstance occurs when subjects are asked to perform a specific number of repetitions of a given task as fast as possible or when subjects walk a given distance at their preferred speed (i.e., self-paced task). This is particularly true when a motor activity is involved [23], but it can also occur when the brain activity is recorded during real-life conditions, such as working activities or navigating through the environment [24,25], or even for brain–computer interfaces [26].

This variability may lead to statistical errors related to the aggregation of data that are not directly comparable [27]. Moreover, most of the commonly used methods for analysis are based on the event-locked or task-locked averaging of specific portions of the continuous fNIRS signal. This is inaccurate in the case of periods of interest with different time durations. In such cases, a time-normalization procedure is needed to match the duration of different segments and concurrently preserve the main features while reducing variability [27], so that results can be comparable between different trials and later across participants.

Temporal normalization of the signal has been previously implemented for EEG data using linear interpolation. In particular, in the case of motor activities, such as walking, the single-trial signals are time-warped so that the different epochs are aligned and analyzed as a percentage of the gait cycle [28,29,30].

As for fNIRS applications, the dynamic time warping (DTW) technique can be exploited in the case of variable latencies in task-induced activities to account for the temporal variation in the alignment between two signals that need to be averaged as an enhanced alternative to the most common method of point-by-point averaging, as in [31]. Furthermore, the DTW algorithm can also be applicable with time series of different lengths, with the goal of finding an optimal alignment between the two given sequences through local compressions and extensions of the temporal axes, with a minimal overall cost [32]. Specifically, the time-axis of one signal is warped so that the maximum coincidence is attained with the other [33]. However, in some cases, the over-stretching or over-compression [34] may not fully preserve the signal shape, leading to a loss of information.

To date, to the best of our knowledge, a time-scale normalization of the fNIRS signal that could address this issue is yet to be developed.

The aim of the present work was therefore to develop a MATLAB function (*TaskNorm.m*) that time-normalizes the fNIRS concentration data, recorded during trials with different durations, as a percentage of the trial by exploiting a spline interpolation method. This step, when applied at the end of the pre-processing pipeline, would allow to obtain fNIRS data of the same length and thus to compare the hemodynamic response function (HRF) of trials of the same task, where there is no a priori defined stimulus/task duration.

The developed function is publicly available and is importable into the HomER3 [9] package, which is one of the most used open-source MATLAB (The Mathworks, Inc., Natick, MA, USA) toolboxes for fNIRS data visualization and analysis. This facilitates the analysis of data recorded during trials with different durations by scientists with low programming skills and contributes to the ongoing standardization process of pipelines for fNIRS data analysis.

In order to test whether, when applying the developed algorithm, fNIRS concentrations were aligned and therefore comparable with each other, we specifically examined experimental fNIRS data that were collected during three different balance control tasks in children with developmental coordination disorder (DCD) and their typically developing (TD) peers. DCD is a neurodevelopmental condition characterized by a delay in acquiring motor skills and poorer execution of coordinated movement, with a significant impact on daily life [35]. DCD occurs in 5–6% of school-aged children, is typically present since early childhood, and cannot be explained by other conditions, such as neurological disorders, and intellectual or visual disabilities [35,36]. Children with DCD struggle with balance in up to 87% of the cases, although the manifestations of these difficulties are very heterogeneous [37]. The brain control mechanisms underlying these alterations are also poorly understood [37,38]. To this end, real-time brain imaging fNIRS recordings during movement can shed light on how the brain interacts with motor skills and balance control [39,40,41]. However, these signals are affected by variability due to the nature of the task being performed, so a further standardization step is required, which can be implemented using the function we proposed here.

In the following sections, detailed descriptions of the algorithm implemented in the *TaskNorm* function (Section 2.1) of the fNIRS data acquisition protocol performed on eight children during balance control tasks and of the signal pre-processing pipeline are provided (Section 2.2). The results concerning the performance and applicability of the function are reported in Section 3.

## 2. Materials and Methods

### 2.1. Algorithm Description

We implemented a MATLAB (R2023a) function *TaskNorm.m* that performs a time-normalization of the concentration data for each repetition of the task, whose duration is converted into a percentage (0–100%) of the trial duration.

**Figure 1:**
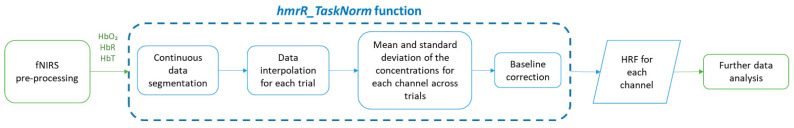
The flowchart of the processing pipeline. The HbO_2_, HbR, and HbT data are given as an input to the *TaskNorm* function, of which each step is highlighted. The output of the function is the time-normalized signals and the HRF for each channel. Abbreviations: fNIRS = functional near-infrared spectroscopy; HbO_2_ = oxyhemoglobin concentration; HbR = deoxyhemoglobin concentration; HbT = total hemoglobin concentration; and HRF = hemodynamic response function.

The main steps of the *TaskNorm* function, and its integration in the processing pipeline, are reported as a flowchart in Figure 1.

The main inputs of the function are the pre-processed concentration signal recorded during the selected experimental task, along with the triggers that delimit each repetition and its duration (stored directly in the raw .snirf files input in the pre-processing step); and the durations (expressed in seconds) of the baseline and the post-task periods, which are defined by the users in the two *deltat* parameters. The first *deltat* value accounts for the duration of the baseline period before the task onset, while the second value accounts for the duration of the post-task period (i.e., after the task offset). The users can choose the appropriate values of these parameters according to both their research question and experimental design. It is also possible not to consider the period after the end of the task (setting the post-task duration to 0 s), making the baseline period the common reference to measure the changes in the hemodynamic activity related to the task.

Once the *deltat* parameters are set, the concentration signal is segmented into several epochs corresponding to the task repetitions, which include the baseline, the effective task, and the post-task periods.

Afterwards, the time-normalization is performed by applying a spline interpolation to each data segment relative to a single task repetition in order to estimate the signal value in a desired and fixed number of points.

We developed two different versions of the time-normalization algorithm. In the first version, the interpolation is performed separately for the baseline, for the task, and for the post-task periods. This allows us to make the three phases distinguishable and to consider the exact onset/end of each task’s repetition. The entire signal is then reassembled for further analysis. Specifically, the baseline and post-task periods are interpolated using a number of samples computed as in Equation (1):

(1) $${\#sample}_{i,j}=C\times {∆t}_{i,j}$$

where ${∆t}_{i,j}\;$ is the *deltat* parameter referring to the baseline period (*i*) or post-task period (*j*), and *C* is a constant value set arbitrarily to have an adequate number of points while at the same time avoiding overfitting.

The number of samples for the task period is calculated as in Equation (2):

(2) #sample=C×m

where *m* is the mean duration of all the repetitions and *C* is a constant value set with the same criterion as above.

In the second version of the function, the whole task repetition is interpolated without distinguishing the phases within it, computing the number of samples as in Equation (2), with *m* being the duration of the three phases considered together. The final percentages related to the onset/end of the task were imposed by taking into account the corresponding values in each repetition, the average of which was selected.

The effect of varying the value of *C*, from *C* = 10 to *C* = 100, was tested by comparing the Euclidean distance between the position of the maximum and minimum peaks of the raw data with the position of the ones after the spline interpolation and the eventual reassembling phase for each repetition. This was used as an error measure of the function. The error becomes stable around *C* = 40, whereas a value of *C* = 90 or 100 would heavily increase the number of samples needed for the interpolation. For these reasons, we chose *C* = 50 as we think this could be an adequate trade-off. However, the function can be easily modified and this constant value can be set based on the experimental data of the specific research (e.g., sampling frequency of the signal).

Regardless of the selected version of the function, both the mean value across repetitions and the standard deviation of HbO_2_, HbR, and HbT concentrations of each channel are then computed to obtain an average HRF for each channel. Next, a baseline correction is applied to the resulting HRF: the mean value of the HRF during only the baseline period is subtracted from the entire HRF to normalize the response, obtaining on average a value of 0 µM for the HbO_2_, HbR, and HbT concentrations during the baseline period.

Furthermore, the developed function also allows the baseline-corrected mean HRF and its standard deviation to be plotted in MATLAB, for both HbO_2_ and HbR concentrations for each channel, and the corresponding concentrations for each repetition (this can be done by setting the input parameter *show* equal to 1; see Supplementary Figure S1).

The function was made compatible with the HomER3 toolbox [9]. Therefore, it is possible to import the function in the HomER3 processing stream, run the stream all at once, and visualize the hemodynamic response function (HRF) outcome directly in the Main GUI, as well as to export the outcome for further analysis outside the HomER toolbox.

### 2.2. Testing of the Algorithm

#### 2.2.1. Participants

The *TaskNorm* function was tested using the data acquired from eight children (age range 8–10 years old), i.e., four children with DCD (mean age: 9.78 ± 0.30, 3 males) and four typically developing children (mean age: 9.02 ± 0.30, 2 males). Prior to inclusion, all children and parents were informed about the methodology and duration of this study. Parents, on behalf of their children, signed official informed consent to participate in the study. This study was conducted according to the guidelines of the Declaration of Helsinki and approved by the Committee for Medical Ethics UZA-UAntwerp (B300201941833).

#### 2.2.2. Experimental Protocol

**Figure 2:**
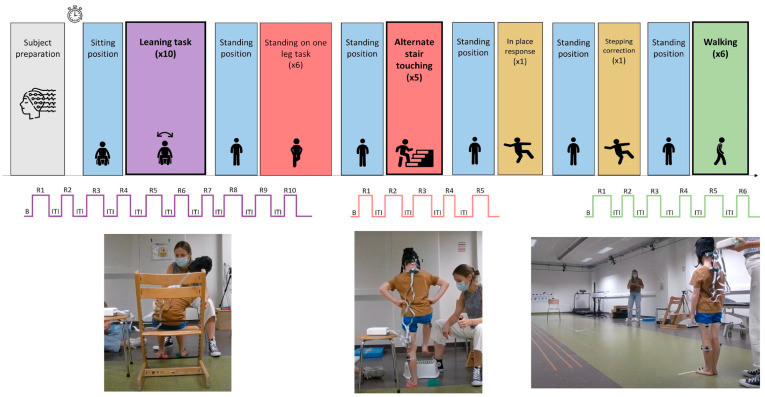
Complete experimental paradigm. For the tasks used for the testing procedure, the number of repetitions and inter-trial intervals and an example photo of a subject performing the task are provided. Abbreviations: B = Initial Baseline; $R_{i}$ = Repetition *i*; and ITI = Inter-trial interval.

All participants performed six different balance tasks in a standardized order, while undergoing fNIRS and electromyography (EMG). For the purpose of this study, EMG data were only exploited to identify the start and the ending of the proposed task by examining muscle activity. The experimental paradigm is presented in Figure 2. The fNIRS and EMG data-recording procedure lasted for approximately 30 min. The proposed tasks were chosen from a physiotherapeutic balance test: the Balance Evaluation Systems Test for Children (Kids-BESTest) [42]. Among the six tasks (described in Table 1), three were selected to test the algorithm. Specifically, we first excluded tasks with only one repetition, as the algorithm requires a minimum of two repetitions to be applied. Among the remaining tasks, we selected one task per section, as presented in the Kids-BESTest: for the ‘Stability in gait’ section, we selected the walking task, which is the most common and analyzed task in the literature; for the ‘Anticipatory postural adjustment’ section, the alternate stair touching task was chosen since it is a more comprehensive task with respect to the others; and for ‘Stability limits’, the leaning task was kept.

The fNIRS raw data collected during the three selected tasks are publicly available at https://doi.org/10.5281/zenodo.10124956, Zenodo.

#### 2.2.3. Signal Acquisition and Pre-Processing

The NIRSport2 device (NIRx Medical Technologies, Berlin, Germany) was used for recording fNIRS data with a sampling rate of 10.27 Hz, with two continuous wavelengths (760 nm and 850 nm), 8 sources, and 8 detectors (dual-tip). The probe configuration was generated using the fNIRS optodes locator decider (fOLD) open source toolbox (Zimeo Morais GA). Specifically, the most specific channels were chosen to represent each region of interest (ROI) (i.e., supplementary motor area—SMA/premotor cortex—PMC and inferior/superior parietal lobule—IPL/SPL brain areas), using an 8-8 optode bundle placed according to the 10-10 international system (Supplementary Figure S2). More precisely, sources were placed at FC3, FCz, FC4, C1, C2, Cp3, CPz, and CP4 positions; whereas detectors were located at FC1, FC2, C3, Cz, C4, CP1, and CP2 channel positions.

Data were acquired using Aurora fNIRS 2021.9 Acquisition Software for Windows (NIRx Medical Technologies, Berlin, Germany). Simultaneously, EMG was recorded using Delsys Trigno™ (Delsys Inc., Natick, USA). As an event marker, to identify start and stop of each trial, an analog input adapter connected to the EMG device (Delsys Inc., Natick, USA) was used.

The fNIRS signal pre-processing was performed using the HomER3 package (v1.72.0) [9] according to the workflow and parameters described in other studies [43,44,45]. Specifically, the raw light intensity was initially converted into optical density with the *hmrR_Intensity2OD* function.

A minimum of 0 and a maximum of 3 channels (Mean = 0.42 SD = 0.88), whose signals were too weak, too strong, or had a high standard deviation with respect to the thresholds defined in [44], were pruned with the *hmrR_PruneChannels* function (setting *dRange*(1) = 5 × 10^−4^; *dRange*(2) = 1; *SNRtresh* = 10; and *SDrange* = [0, 45]) and then discarded from further analysis.

Motion artefacts correction was performed using a combination of a 5-s moving average filter and a discrete wavelet transform was applied to every channel data series exploiting the *hmrR_MovingAverage* and *hmrR_MotionCorrectWavelet* functions (setting the *interquartile range* to 0.1, as in [46]), and as performed in [43]. The remaining motion artefacts were then detected using the *hmrR_MotionArtifactByChannel* function (setting *tMotion* = 1; *tMask* = 1; *STDEVthresh* = 50.0; and *AMPthresh* = 0.60).

Moreover, a band-pass filter (*hpf* = 0.01; *lpf* = 0.2, cut-off frequencies expressed in Hz) was used to remove instrumental and physiological noises with the *hmrR_BandpassFilt* function.

The optical density data were converted to hemoglobin concentrations (HbO_2_, HbR, and HbT) applying the Modified Beer–Lambert Law (MBLL), which is implemented in the *hmrR_OD2Conc* function (*ppf* = [1.8, 1.8]).

Finally, the *hmrR_TaskNorm* function (*deltat* = [2, 3]) was used to obtain the average HRF for each analyzed task. We set the duration of the baseline to 2 s, which can be considered an adequate period of time to obtain an accurate average of the baseline signal, while the post-task duration was imposed at 3. The outcome of the *hmrR_TaskNorm* function can be used for further analyses (e.g., compare HRF of different channels or groups of subjects, as done in Section 3.2).

#### 2.2.4. Statistical Analysis

Both versions of the algorithm were applied to the three tasks to test their working principle. A quantitative validation of the function performance in preserving the shape of the raw signal was performed by comparing with non-parametric paired tests the amplitude and the time of occurrence of maximum and minimum peaks between the two signals.

In addition, for each subject and repetition, the onset and offset values were stored (see Supplementary Materials) and the difference between the outcomes of the two versions of the function was calculated to compare the error made by the second one.

Shapiro–Wilk normality test was performed to verify the distribution of the resulting data. We then investigated the differences between the performances of the two algorithms in terms of accuracy in identifying onset and offset percentages with respect to the entire task duration by means of a Wilcoxon signed-rank test, according to the normality test outcome. The One Sample *t*-test was applied to the error data to statistically compare the mean of the observed data to the assumed zero mean. Furthermore, the two codes were compared in terms of smoothness of signals with a Wilcoxon signed-rank test. The standard deviation of the first derivative of each task repetition was computed as an index of signal smoothness.

For all statistical tests, significance level was set at *p* < 0.05.

## 3. Results

### 3.1. Results of the TaskNorm.m Function

**Figure 3:**
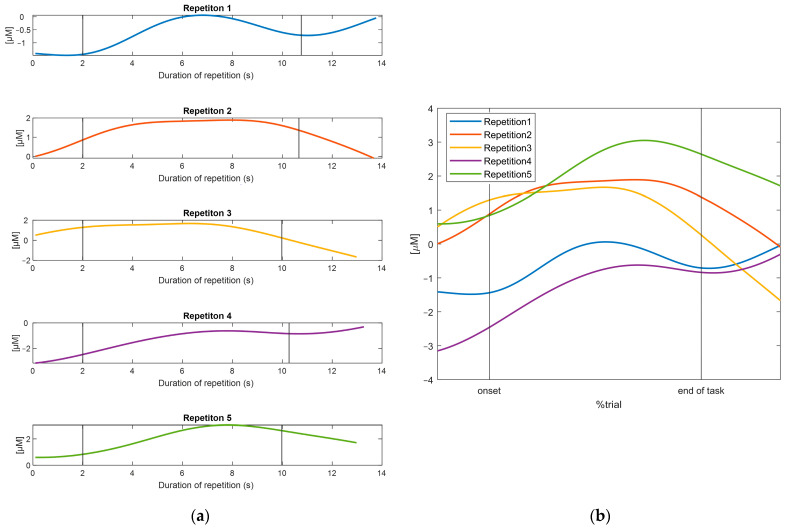
(**a**) The concentration data for each repetition before interpolation and (**b**) the concentration data following the time-normalization procedure. The figures are referring to the HbO_2_ data recorded by one channel for one subject performing one task, as an example.

The example provided in Figure 3 shows how the time-scale normalization with the spline interpolation preserved the original shape of the data, while aligning the time scale. Statistical tests on pre-post interpolation differences at the time of occurrence of the characteristic points yielded no statistically significant differences (*p* > 0.05), whereas significant differences are present when considering the amplitude of such points (*p* < 0.01). However, the mean difference between the raw and interpolated signals of the amplitude of the maximum peaks and minimum peaks were 1.61 × 10^−4^ µM and 1.62 × 10^−4^ µM respectively, which we assume to be acceptable.

Time-normalization, thus, allowed for an effective comparison, and a subsequent averaging, across different repetitions of the tasks and consequently between subjects.

Table 2 displays the percentage of onsets and offsets in relation to task duration, along with the corresponding standard deviation, for each subject and task as obtained by the two different methods. For the sake of simplicity, the table shows only the mean values over the repetitions of each task, while the statistical tests described in Section 2.2.4 were performed on the data presented in Supplementary Table S1. In the first version, the values correspond exactly to the beginning and end of the task (this is why the resulting standard deviations are zero), since the three phases were kept distinguishable, while in the second case, the values obtained are only an estimation. The statistical analysis yielded a significant difference between the two functions only in the identification of the offsets (*p* < 0.01) and the related error values (*p* < 0.001).

As for the smoothness of the signals, the results of the performed test showed statistically significant differences between the two versions (*p* < 0.01) for each channel and for both the HbO_2_ and HbR concentration signals.

Both versions of the *TaskNorm.m* are publicly available on Zenodo (https://doi.org/10.5281/zenodo.10043158). Furthermore, the first version of the function has been merged into the HomER3 master branch on GitHub, so it is now available as part of the HomER3 toolbox at https://github.com/BUNPC/Homer3/tree/master/FuncRegistry/UserFunctions (accessed on 21 December 2023). The aim of the present work is thus to describe, for the first time, to a possible user of the function, its rationale, the working principle, and its applicability in real data analysis, describing also the possible differences between the two versions.

**Figure 4:**
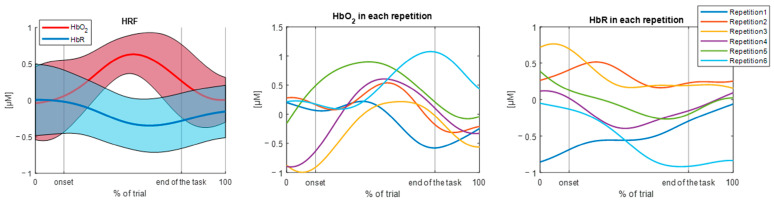
Example of the output of the function when setting *deltat* = [2, 3]. The output is related to a single channel during the walking task, for a single subject. Mean hemodynamic responses (and standard deviation area), time-normalized HbO_2_ concentrations for each repetition, and time-normalized HbR concentrations for each repetition are shown. Abbreviations: HRF = hemodynamic response function; HbO_2_ = oxyhemoglobin concentration; and HbR = deoxyhemoglobin concentration.

**Figure 5:**
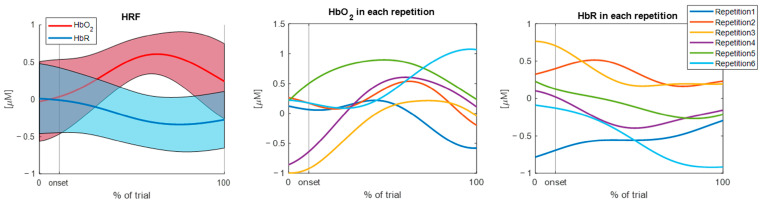
Example of the output of the function when setting *deltat* = [1, 0]. The output is related to a single channel during the walking task, for a single subject. In this case, the post-task period has not been considered. Mean hemodynamic responses (and standard deviation area), time-normalized HbO_2_ concentrations for each repetition, and time-normalized HbR concentrations for each repetition are shown. Abbreviations: HRF = hemodynamic response function; HbO_2_ = oxyhemoglobin concentration; and HbR = deoxyhemoglobin concentration.

Figure 4 and Figure 5 show two examples of the output of the function, when setting the *show* parameter to 1, related to a single channel for one subject performing the walking task. Specifically, the effect of the *deltat* parameters is highlighted: Figure 4 displays the hemodynamic response with a 2-s baseline and a 3-s post-task period (*deltat* = [2, 3]), whereas Figure 5 demonstrates the possibility of adjusting the baseline duration to normalize the signal and not considering the post-task period (*deltat* = [1, 0]). In both cases, the mean baseline-corrected HRF and its standard deviation are reported for both the HbO_2_ and HbR signals, as well as the concentration data for each repetition. The present function provides the same output for each channel.

Once the function is imported into the HomER processing stream, the HRF outcome is displayed in the Homer3 MainGUI (see Supplementary Figure S3) and may be further analyzed using the other available tools in the HomER package [9].

### 3.2. Results of the Testing

Once the HRF had been extracted for each subject and task, it was possible to compute the mean HRF for the two groups for the selected channel. Both versions of the algorithm were applied to the three tasks to test their working principle. For the sake of simplicity and in order to show the results derived from the use of both versions, the outcome HRF during the alternate stair touching task and the leaning task obtained with the first version of the function and the walking task HRF derived with the second version are presented in the current work.

**Figure 6:**
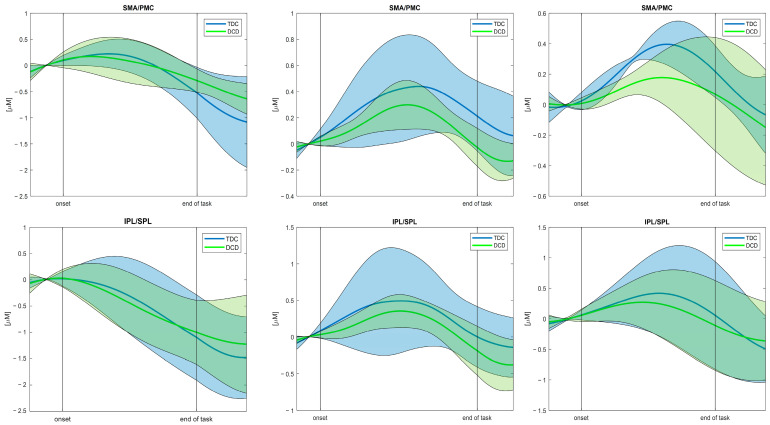
Example of the hemodynamic response (mean and standard deviation area) related to HbO_2_ concentrations averaged across subject and channel clusters (up: SMA/PMC regions of interest, down: IPL/SPL regions of interest), divided into the three tasks: **First column**) Alternate stair touching task; **Second column**) Leaning task; and **Third column**) Walking task. An increasing trend during task execution and a subsequent decrease towards and during the post-task period can be observed. Abbreviations: SMA = supplementary motor area; PMC = premotor cortex; IPL = inferior parietal lobule; and SPL = superior parietal lobule.

Figure 6 depicts the HRF outcomes for the different tasks, referring to HbO_2_ concentrations, averaged across DCD and TD children and across the defined channel ROIs. As for the baseline period, a reduced standard deviation can be noticed across all tasks and all the ROIs due to the baseline-correction procedure that bounds that portion of the signal in a range around 0 µm. During the effective task execution period, a task-induced increase can be generally observed in the mean HRF, relative to the HbO_2_ concentration. On the other hand, a general decreasing trend in the hemodynamic responses can be observed in the post-task periods that can be considered a resting phase with lower brain activation.

**Figure 7:**
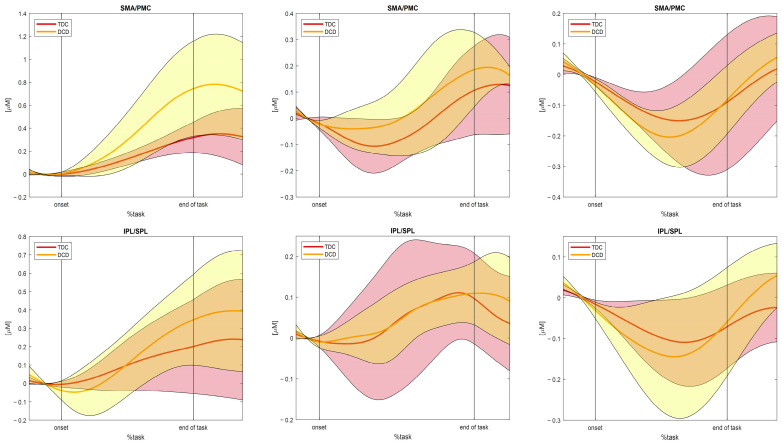
Example of the hemodynamic response (mean and standard deviation area) related to HbR concentrations averaged across subject and channel clusters (up: SMA/PMC regions of interest, down: IPL/SPL regions of interest), divided into the three tasks: **First column**) Alternate stair touching task; **Second column**) Leaning task; and **Third column**) Walking task. A decreasing trend during task execution and a subsequent increase towards and during the post-task period can be observed, opposite to the HbO_2_ behaviour. Abbreviations: SMA = supplementary motor area; PMC = premotor cortex; IPL = inferior parietal lobule; and SPL = superior parietal lobule.

The same averaging procedure was performed for the HRF related to the HbR concentrations (Figure 7). A general opposing trend can be found in the HbR concentrations with respect to the HbO_2_ concentrations during the different tasks. In particular, during the execution of the task, a decrease in the HbR concentration and a subsequent increase towards and during the post-task period can be observed. HbR concentrations during the baseline periods can be interpreted in the same way as for the HbO_2_.

Neither the group nor the task analyzed had an impact on the algorithm’s outcomes.

## 4. Discussion

The present study introduces a novel function for the time-normalization of hemodynamic responses recorded during trials in which there is no a priori defined task duration. This is particularly true for experimental designs that include motor tasks that can be performed at self-paced conditions. Indeed, in such cases it is not possible to compare data recorded during either different repetitions of the same task or between different subjects. For this reason, the function interpolates the processed data with a fixed number of samples, which are then expressed as a function of the percentage of the trial duration. This allows us to successively apply the standard procedure of analyzing fNIRS data (i.e., averaging across trials to obtain the mean hemodynamic responses) without the need of advanced programming skills.

The *TaskNorm* function operates with the same fundamental concept as the HomER3 function *hmrR_BlockAvg* [9], computing the mean baseline-corrected HRF of the concentrations of each repetition of the task. However, the original *hmrR_BlockAvg* function is not applicable in the experimental conditions as outlined above, since the inputs of this function are the baseline period before the task onset and a fixed interval period after the task onset across all trials. The variability in the duration of each repetition is therefore not considered, leading to incorrect averaging and comparisons. Hence, there is a need to develop a new function to address this issue.

Other techniques, such as the DTW algorithm, are exploited in the field of fNIRS analysis [31] to align one signal to a second time series, which can also be recorded during trials of different durations, through local compressions and extensions of the temporal axes. However, the stretching of the signals may lead to a modification of the original shape, which would not be preserved. Furthermore, DTW-based averaging can be realized by aligning the data to a reference signal (which we do not have) or the average must be obtained sequentially in a pairwise manner, with a risk of error propagation. In these cases, the temporal alignment is performed with respect to the signal of reference, which would result in an incorrect temporal representation of the final HRF. For these reasons, a direct quantitative comparison of the performance of the DTW with our function is not possible.

The present work aimed to address the aforementioned issues by implementing the *TaskNorm* function, which was designed with the primary purpose of normalizing the data to ensure a proper comparison. Two versions of the *TaskNorm* function were developed with different modalities of data interpolation. In the first version, the three phases of the task (i.e., baseline, task duration, and post-task duration) are separately interpolated, and then the signal is reassembled, while in the second version, the function interpolates the signal related to the entire task. The first version allowed the onset and the end of the task to be accurately identified as a percentage of the trial, while statistical analyses show that the second version made a significant error in identifying the offset. Thus, this first version can be particularly useful when the experimental design requires a higher temporal resolution, as in the case of rapid event-related designs or when fNIRS is combined with EEG recordings. The first version of the algorithm could also be preferred when exploring plausible between-subject differences specifically related to either the “preparation/planning phase” of the response or the post-task recovery, or when interested in analyzing individual intra-trial differences. Conversely, the signals obtained with the latter version have a significantly lower smoothness index, thus resulting in a smoother signal. This version seems therefore more appropriate for research focusing on the overall hemodynamic response throughout the entire trial.

Both versions of the function were tested on fNIRS data recorded in eight participants (four children with DCD and four TD children) during three different tasks (alternate stair touching, leaning, and walking). Participants were asked to perform each task for a fixed number of times at their own pace, so that no fixed trial duration was imposed.

The present results show that the algorithm correctly allows comparisons and averaging of data from each channel across multiple repetitions of the same task, even if they have different durations, because of the time-normalization performed.

Thereafter, hemodynamic responses for each channel were averaged across subjects and then across SMA/PMC and IPL/SPL brain ROIs for each task. The resulting HRFs are consistent with the previous literature [17,47], as a general increase in cortical activity that is attributable to task performance was expected, whereas an opposing trend characterizes the HbR responses. Nevertheless, the small sample size represents a limitation of this study, thus it is important to acknowledge that these responses can be analyzed only qualitatively, as they are the result of a comparison on a limited number of subjects. Indeed, the purpose of this study is to test the applicability of the implemented algorithm to obtain a mean HRF value across repetitions for each subject (which allows the subsequent average computation across subjects), rather than study the brain activation elicited by the different tasks or the differences between the two groups.

The final outcome of the function is independent either from the task or from the pre-processing pipeline applied to the data, since the only parameters needed are the continuous fNIRS signal of the task considered, and the durations of the baseline and the post-task periods (both parameters are set by the users). We are aware that because of our selection of the *deltat* parameters, an overlap between the HRF from one repetition and one from a successive repetition may occur. However, our purpose was to present the entire functionality of the *TaskNorm* function and statistically evaluate all the differences between the two versions (i.e., trial phase segmentation, onset/offset identification). For this reason, we decided to perform the analysis while also considering the post-task period. However, the user can select the appropriate *deltat* values for the experimental protocol in order to obtain proper results. This ensures the generalizability and flexibility of the function for different experimental designs, including motor or cognitive task analysis.

The present function introduced a time-normalization step in an already existing and widely used procedure (i.e., block-average analysis). This was done to enable this analysis to be performed correctly even under conditions where it could not be (e.g., varying tasks duration). Future developments envisage the possible integration of this step into other analysis techniques that do not take into account different trial durations.

Finally, future prospects will focus on possible applications of the algorithm to a larger number of subjects but also to different populations, such as healthy or neurologically impaired individuals, as well as pediatric and adult ages. Participants may perform self-paced motor/balance tasks but also cognitive or in real-life conditions tasks, such as working activities. Different brain regions can be investigated (such as the frontal, parietal, or temporal areas) also enabling quantitative assessments of group-related or task-related differences.

## 5. Conclusions

The present work responds to the need for a method suitable for fNIRS analysis to compare data across different trials and subjects in studies where task trials have variable durations. To this end, we developed the *TaskNorm.m* function, which allows rescaling of the time-axis in each trial to a common reference duration set to a percentage of the trial duration, with a spline interpolation method. The novelty of the current study lies mainly in the application of the fNIRS data analysis, since time-normalization techniques for fNIRS data in such conditions are yet to be implemented, rather than in the methodological aspects, as it exploits an already existing methodology.

The function is structured so that it is readable and importable in the HomER processing stream by the user, but is also available as part of the HomER3 package [9], which is a toolbox developed and distributed to facilitate the processing of the fNIRS signal, which still lacks a well-defined and standardized procedure due to the relative novelty of this imaging technique [8].

Moreover, the function is already available on the Zenodo platform, and both versions can be used independently, without being imported into the HomER package. This makes them accessible to those who do not use this package or are merely interested in the time-normalization procedure. Additionally, the main aspects of the function can be easily converted to other programming languages (e.g., C++ or Python), so that is widely applicable to different settings and experimental research.

Therefore, we feel that the function proposed here could be a further step in contributing to the ongoing process of developing a standardized signal analysis pipeline for fNIRS data.

## Figures and Tables

**Table 1:** Brief descriptions of the tasks proposed in the experimental protocol. Bold text indicates the tasks selected for the testing of the algorithm.

Task			Baseline/Rest Position		Repetitions	
Kids-BEST Test Section	Name	Explanation	Description	Intra-Trial Mean Duration (SD) [s]	N	Mean Duration (SD) [s]
Anticipatory postural adjustment	**Alternate stair touching**	The child taps with their feet on a stool in front of them alternately with the left and right foot as fast and as controlled as possible.	Standing on two feet	12.70 (4.11)	5 times	7.81 (1.46)
Stability limits	**Leaning left and right while seated**	The child leans as far and as stable as possible sidewards while seated, without falling and keeping their feet on the ground. Arms are crossed at the chest.	Sitting	12.59 (12.16)	10 times	12.87 (2.25)
Stability in gait	**Walking**	The child walks 6 m over level ground as fluently as possible.	Standing on two feet	11.3 (6.91)	6 times	6.60 (1.30)
Anticipatory postural adjustment	Standing on one leg	The child stands on one leg for as long as possible. This exercise is then repeated with the other leg.	Standing on two feet	60.56 (22)	6 times	18.88 (11.21)
Reactive postural responses	In place response—backward	The therapist holds the child, who resists. When the therapist suddenly releases the child, he/she should keep balancewithout taking a step.	Standing on two feet	-	1 time	0.1 (0)
Reactive postural responses	Compensatory stepping correction—backward	The child leans beyond theirbackward limits against the therapist’s hands. When the therapist suddenly releases the child, he/she should be able to avoid falling, perhaps even taking a step.	Standing on two feet	-	1 time	0.1 (0)

**Table 2:** Difference between first and second version in identifying onset and offset. Mean values (and standard deviation) with respect to %trial are reported for each subject and task.

			First Version		Second Version	
Task	ID Code	Mean Duration (SD)	%Mean Onset (SD)	%Mean Offset (SD)	%Mean Onset (SD)	%Mean Offset (SD)
Walking	DCD_1	8.38 (1.26)	15.15 (0)	76.97 (0)	14.95 (1.26)	76.89 (2.02)
Walking	DCD_2	5.16 (0.94)	19.72 (0)	70.02 (0)	19.64 (1.78)	69.54 (2.79)
Walking	DCD_3	6.76 (0.47)	16.42 (0)	75.04 (0)	17.03 (0.69)	73.79 (1.07)
Walking	DCD_4	6.27 (0.66)	17.92 (0)	72.76 (0)	17.85 (1.04)	72.53 (1.67)
Walking	TD_1	6.25 (0.5)	17.92 (0)	72.76 (0)	17.67 (0.75)	72.56 (1.16)
Walking	TD_2	5.34 (0.37)	19.72 (0)	70.02 (0)	19.24 (0.7)	70.14 (1.09)
Walking	TD_3	6.31 (0.42)	17.92 (0)	72.76 (0)	17.67 (0.69)	72.86 (1.03)
Walking	TD_4	8.32 (0.35)	15.15 (0)	76.97 (0)	14.95 (0.34)	76.89 (0.55)
Alternate stair touching	DCD_1	10.21 (0.96)	13.12 (0)	80.05 (0)	13.08 (0.77)	79.71 (1.24)
Alternate stair touching	DCD_2	6.89 (1.07)	16.42 (0)	75.04 (0)	16.69 (1.39)	74.12 (2.2)
Alternate stair touching	DCD_3	7.63 (0.93)	15.15 (0)	76.97 (0)	15.87 (1.2)	75.5 (1.86)
Alternate stair touching	DCD_4	9.09 (0.99)	14.06 (0)	78.62 (0)	14.16 (0.93)	78.11 (1.5)
Alternate stair touching	TD_1	6.89 (1.17)	16.42 (0)	75.04 (0)	16.86 (1.61)	73.96 (2.61)
Alternate stair touching	TD_2	6.24 (0.28)	17.92 (0)	72.76 (0)	17.85 (0.49)	72.5 (0.79)
Alternate stair touching	TD_3	7.23 (0.28)	16.42 (0)	75.04 (0)	16.36 (0.3)	74.79 (0.52)
Alternate stair touching	TD_4	8.3 (0.37)	15.15 (0)	76.97 (0)	14.95 (0.43)	76.89 (0.66)
Leaning	DCD_1	12.87 (1.22)	10.93 (0)	83.39 (0)	11.23 (0.8)	82.65 (1.23)
Leaning	DCD_2	11.12 (1.43)	12.3 (0)	81.3 (0)	12.39 (1.2)	80.85 (1.9)
Leaning	DCD_3	13.17 (1.44)	10.93 (0)	83.39 (0)	11.01 (0.85)	82.98 (1.32)
Leaning	DCD_4	11.45 (1.05)	12.3 (0)	81.3 (0)	12.14 (0.74)	81.23 (1.15)
Leaning	TD_1	13.28 (1.23)	10.93 (0)	83.39 (0)	10.9 (0.69)	83.09 (1.08)
Leaning	TD_2	12.65 (1.21)	10.93 (0)	83.39 (0)	11.35 (0.78)	82.54 (1.22)
Leaning	TD_3	11.37 (0.79)	12.3 (0)	81.3 (0)	12.14 (0.6)	81.23 (0.89)
Leaning	TD_4	17.09 (2.45)	8.93 (0)	86.42 (0)	9.1 (0.94)	85.99 (1.48)

## Data Availability

Publicly available datasets were analyzed in this study. This data can be found here: https://doi.org/10.5281/zenodo.10124956, Zenodo. Both versions of *TaskNorm.m* are publicly available on Zenodo at https://doi.org/10.5281/zenodo.10043158. The first version of the function is available as part of the HomER3 toolbox at https://github.com/BUNPC/Homer3/tree/master/FuncRegistry/UserFunctions (accessed on 21 December 2023).

## Supplementary Materials

The following supporting information can be downloaded at https://www.mdpi.com/article/10.3390/s25061768/s1, Figure S1: Screenshot of the Processing Stream GUI with the parameters set for the presented analysis; Figure S2: Clustering of channels in SMA/PMC (green area) and IPL/SPL (yellow area) regions of interest. Abbreviations: SMA = supplementary motor area; PMC = premotor cortex; IPL = inferior parietal lobule; and SPL = superior parietal lobule; Figure S3: Homer3 MainGUI. Example of hemodynamic response (HRF) outcome (oxyhemoglobin-HbO_2_ and deoxyhemoglobin–HbR concentrations). Table S1: Difference between first and second version in identifying onset and offset.

## Abbreviations

The following abbreviations are used in this manuscript:

fNIRS	functional near-infrared spectroscopy
HbO_2_	oxyhemoglobin
HbR	deoxyhemoglobin
HT	total hemoglobin
fMRI	functional magnetic resonance imaging
EEG	electroencephalography
DTW	dynamic time warping
DCD	developmental coordination disorder
TD	typically developing
HRF	hemodynamic response function
EMG	electromyography
fOLD	fNIRS optodes locator decider
SMA	supplementary motor rea
PMC	premotor cortex
IPL	inferior parietal lobule
SPL	superior parietal lobule
ROI	regions of interest
