# Supplementary figures and images for: Time-Normalization Approach for fNIRS Data During Tasks with High Variability in Duration
Source: Sensors (Basel). 2025 Mar 12;25(6):1768. doi: 10.3390/s25061768 (PMC11945418; doi:10.3390/s25061768)

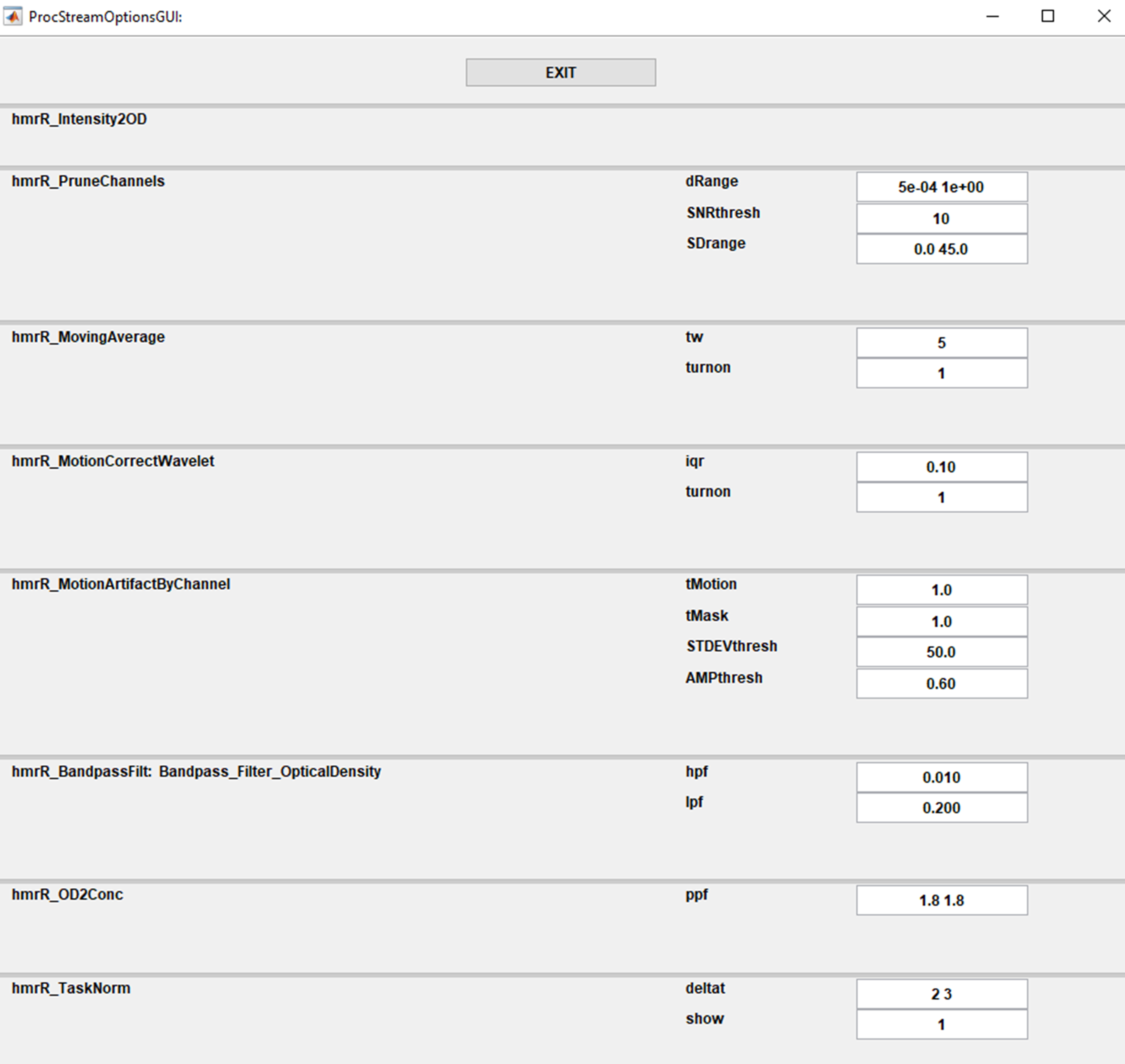

Supplement: Supplementary file 1 [file sensors-25-01768-s001.zip › Supplementary Figure S1.png]

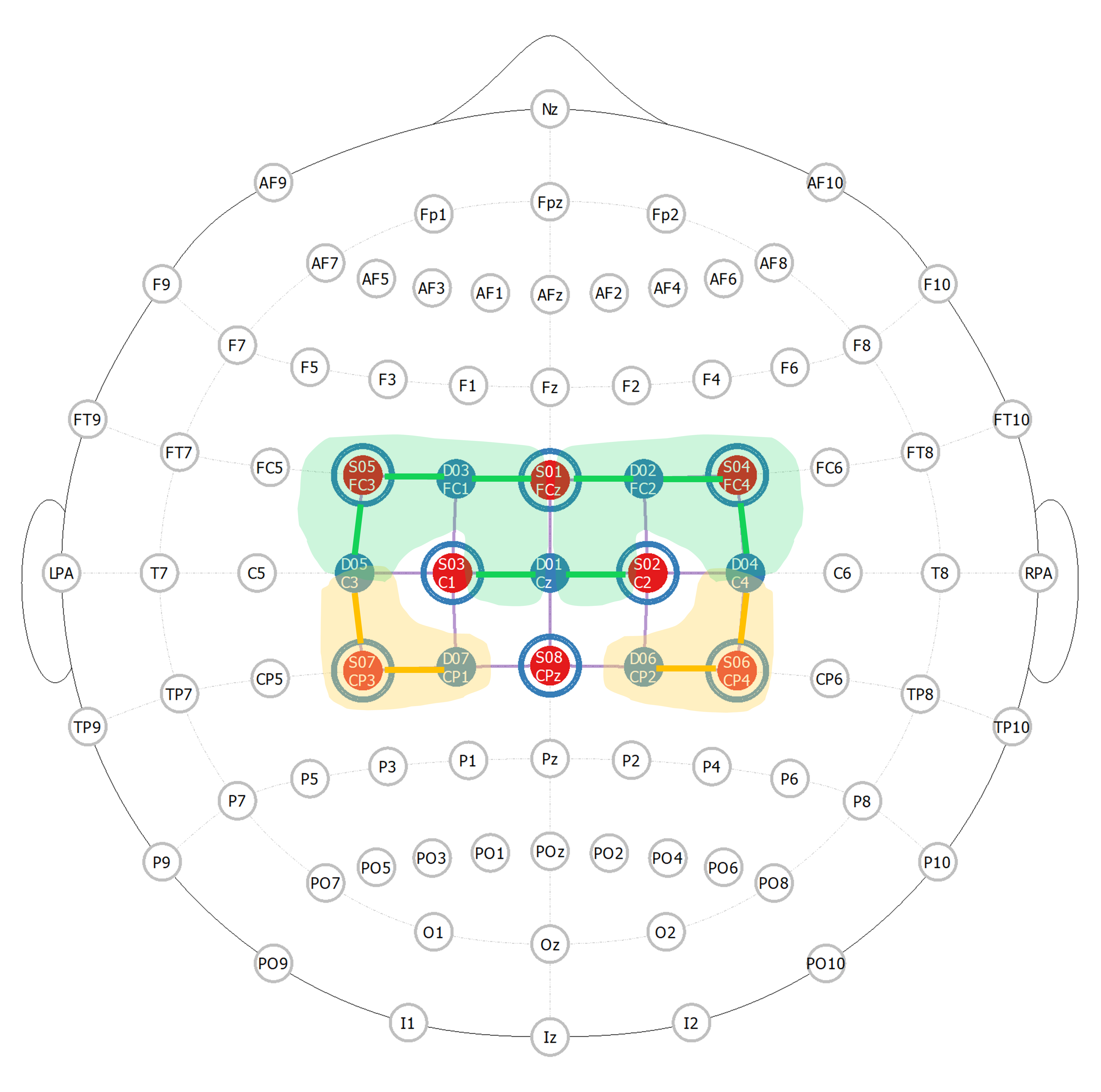

Supplement: Supplementary file 1 [file sensors-25-01768-s001.zip › Supplementary Figure S2.png]

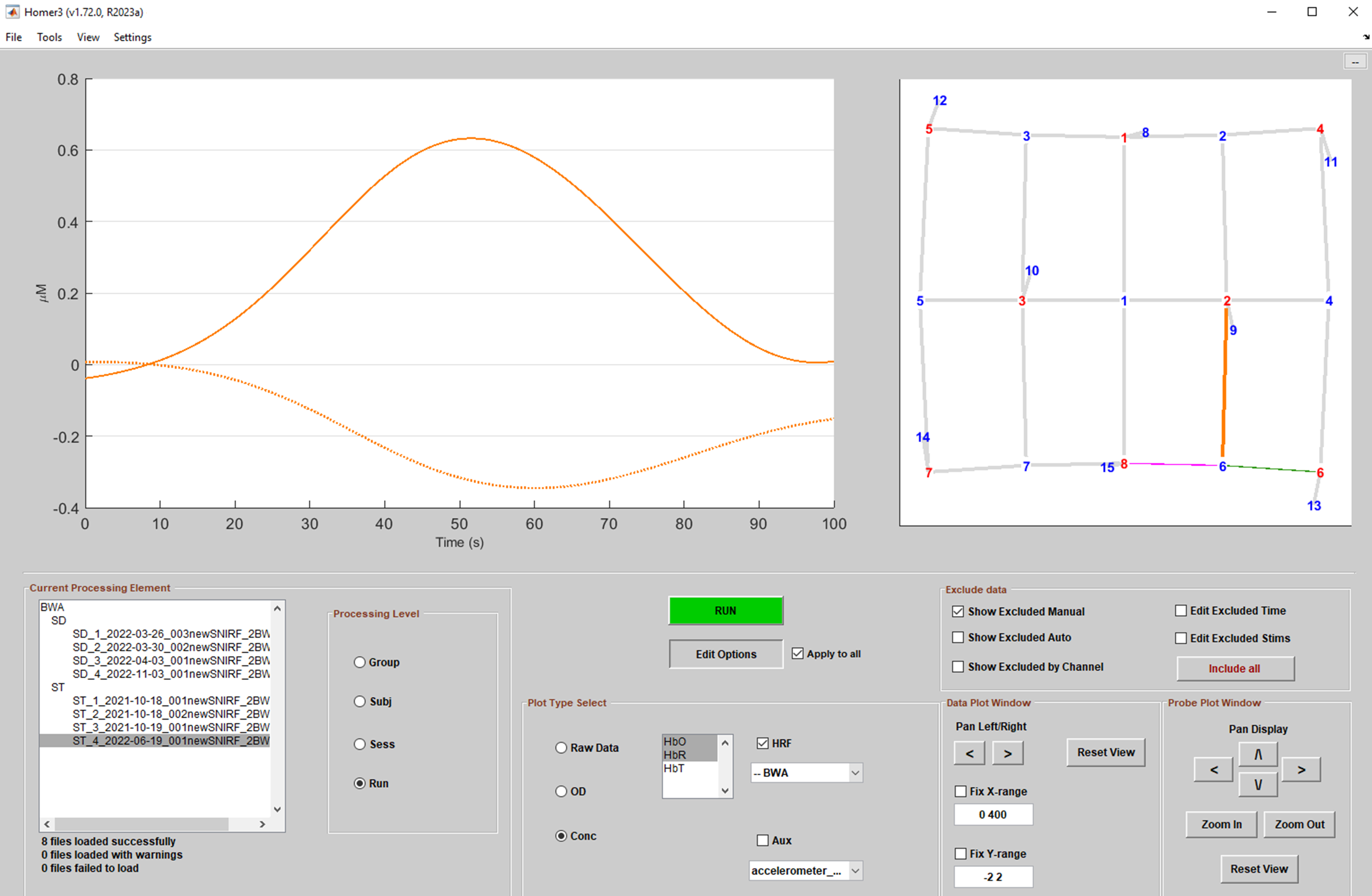

Supplement: Supplementary file 1 [file sensors-25-01768-s001.zip › Supplementary Figure S3.png]
